# Non pharmacological high-intensity ultrasound treatment of human dermal fibroblasts to accelerate wound healing

**DOI:** 10.1038/s41598-021-81878-1

**Published:** 2021-01-28

**Authors:** Jeong Yu Lee, Dae-Jin Min, Wanil Kim, Bum-Ho Bin, Kyuhan Kim, Eun-Gyung Cho

**Affiliations:** Basic Research & Innovation Division, R&D Unit, AmorePacific Corporation, 1920 Yonggu-daero, Giheung-gu, Yongin-si, Gyeonggi-do Republic of Korea

**Keywords:** Biological techniques, Biotechnology, Cell biology, Health care

## Abstract

Inspired by the effectiveness of low-intensity ultrasound on tissue regeneration, we investigated the potential effect of short-term high-intensity ultrasound treatment for acceleration of wound healing in an in vitro wound model and dermal equivalent, both comprising human dermal fibroblasts. Short-term ultrasound of various amplitudes significantly increased the proliferation and migration of fibroblasts and subsequently increased the production of the extracellular matrix components fibronectin and collagen type I, both of which are important for wound healing and are secreted by fibroblasts. In addition, ultrasound treatment increased the contraction of a fibroblast-embedded three-dimensional collagen matrix, and the effect was synergistically increased in the presence of TGF-β. RNA-sequencing and bioinformatics analyses revealed changes in gene expression and p38 and ERK1/2 MAPK pathway activation in the ultrasound-stimulated fibroblasts. Our findings suggest that ultrasound as a mechanical stimulus can activate human dermal fibroblasts. Therefore, the activation of fibroblasts using ultrasound may improve the healing of various types of wounds and increase skin regeneration.

## Introduction

Ultrasound consists of sound waves with frequencies above the upper audible limit of human hearing (> 20 kHz), and propagates through matter. Its therapeutic application has expanded significantly for indications ranging from cancer therapy to thrombolysis^1,2^. Several studies have demonstrated that ultrasound waves can induce mechanical motion of molecules in periodically alternating phases of compression and rarefaction, thereby stimulating tissue regeneration by transmitting mechanical energy^3–6^. In particular, mechanical effects generated by low-intensity ultrasound (< 3 W cm^−2^) can induce changes in proliferation, differentiation, immunological response and gene expression through the activation of intracellular signaling pathways such as the integrin-mediated, mTOR, Rho/ROCK/Src/ERK, NF-κB, and PI3 kinase pathways in various cell types^7–12^. In contrast to low-intensity ultrasound, the cellular effects and mechanisms associated with high-intensity ultrasound (HIUS) have not been widely investigated in human cells in vitro due to the potentially damaging effects of high energy. Nonetheless, HIUS was used as an effective reprogramming strategy to generate stem or progenitor cells from human fibroblasts by inducing the cellular uptake of biomolecules via cavitation^13^. Previous studies have suggested that HIUS, particularly short-time HIUS, may have applications in regenerative medicine by stimulating cells without inducing cell death^13–15^.

Wound healing is a complex process that involves several cell types and sequential repair responses, such as blood clotting, inflammation, proliferation, migration, contraction and remodeling, in damaged tissue^16–18^. Recapitulated cellular migration predominantly occurs via local wound signals that lead to changes in the transcriptome. Cells, particularly fibroblasts, undergo epigenetic reprogramming during wound repair^19,20^. Distinct fibroblast lineages, including dermal fibroblasts, fibrocytes and dermal papilla, are associated with different repair stages. Fibroblasts contribute to fibroplasia and granulation by depositing new collagen matrix and fibronectin, and to wound closure through their contractility revealing that fibroblast activation, characterized by migration and proliferation, are important features of wound healing^17,21^. Several factors, including mechanical properties and oxygen levels, have been shown to activate fibroblasts in wound repair^22,23^.

In this study, short-term HIUS treatment was sufficient to promote the proliferation and migration of fibroblasts and to increase the production of fibronectin and collagen. Based on RNA-sequencing and bioinformatics analyses, mitogen-activated protein kinase (MAPK) signaling pathways were activated at early time points, while the upregulation of cell proliferation-related genes was observed at later time points. Furthermore, activated human dermal fibroblasts facilitated the contraction of a fibroblast-embedded three-dimensional collagen matrix. This novel approach may be developed for tissue regeneration in a variety of medical settings.

## Materials and methods

### High-intensity ultrasound stimulation

Human dermal fibroblasts derived from the skin of a 33-year old adult (Female) were purchased (Cat. No. CC 2511, Lonza, Basel, Switzerland) and cultured in DMEM supplemented with 10% fetal bovine serum (FBS) (Lonza, Basel, Switzerland). The cells were seeded at a density of 5 × 10^4^ per well of a 24-well plate and incubated for 24 h. The cells were stimulated with ultrasound at various amplitudes (10 to 15%) for 10, 30, or 60 s under serum-free DMEM using a 0.5-inch diameter ultrasound transducer (Branson 450, Emerson, MO), which generated continuous ultrasound with a frequency of 20 kHz (spatial average: 400 W cm^−2^). The bottom of the culture plate was held with 1 cm thick sponge. The cells were incubated in DMEM with 10% FBS for 10 min to 120 h according to the purpose of analyses.

### Cell viability and proliferation assay

Cells seeded at a density of 5 × 10^4^ per well of a 24-well plate and incubated for 24 h were stimulated with ultrasound at various amplitudes of 10 to 15% for 10, 30, or 60 s in serum-free DMEM. After the stimulation, cells were incubated in DMEM with 10% FBS up to 120 h. The viability was measured using Cell counting kit-8 (CCK 8) assay (Dojindo, Kumamoto, Japan), and cell numbers over time were counted using Countess II automated cell counter (Thermo Fisher Scientific, Waltham, MA, USA). Cell viability and proliferation assay were performed in triplicate independently.

### Flow cytometry analysis

The cells were treated with ultrasound at 10, 12, or 15% amplitude for 10 s and incubated in DMEM with 10% FBS for 24 h. The cells were collected by centrifugation at 1100 rpm for 5 min and washed twice with PBS. After incubation with FITC-conjugated annexin V and propidium iodide for 15 min using a commercial kit (TACS Annexin V-FITC Apoptosis Detection Kit, R&D Systems, Minneapolis, MN, USA), stained cells were analyzed using a FACSCalibur flow cytometer (BD Biosciences, San Jose, CA, USA) for cell-cycle profile.

### Environmental scanning electron microscope (ESEM) observation

Fibroblast was seeded in 4-well chamber slide (Thermo Fisher Scientific) and incubated for 24 h. After treatment with ultrasound at 12% amplitude for 10 s, cells were incubated in DMEM with 10% FBS for 2 h or 24 h. After incubation, cells were fixed with 4% formaldehyde solution for 4 h at 4 °C, and then washed with 1:10 diluted PBS. Environmental Scanning Electron Microscope (FEI XL30, Philips, NV, USA) images were taken with specimens that had not been dried after removing PBS.

### In vitro scratch wound assay

Human fibroblast was seeded at a density of 5 × 10^4^ per well of a 6-well plate and maintained in 10% FBS containing DMEM for 24 h. The cell monolayer was scraped in a straight line with a sterile 200 µL tip. The cells were treated with ultrasound at different amplitudes (10, 12 or 15%) for 10 s or medium alone for control in serum-free DMEM, and then the media were removed. The cells were incubated in DMEM with 10% FBS in the presence of 5 μg mL^−1^ mitomycin-C for 48 h. The wells were photographed every 24 h, and the analysis was performed in triplicate.

### Transwell migration assay

The cells (1 × 10^4^ per well) were counted and spread in a 24-well plate and allowed to float in serum-free DMEM. The cells were treated with ultrasound at different amplitudes (10, 12 or 15%) for 10 s and the same number of cells were transferred to each transwell (8.0 μm of pore size, Corning Inc, Corning, NY, USA) in a 24-well plate and then incubated in DMEM with 10% FBS in the presence of 5 μg mL^−1^ mitomycin-C for 12 or 24 h. The cells passed through the membranes were fixed and stained. The migrated cells were counted under optical microscopy. The migration assay was performed in triplicate of independent experiments (n = 3).

### Assay for procollagen type I carboxyterminal peptide (PIP) and fibronectin levels

The cells were treated with ultrasound at different amplitudes (10, 12, or 15%) for 10 s in serum-free DMEM conditions and incubated in DMEM with 10% FBS for 0.5, 24, or 48 h. The secreted PIP or fibronectin levels in the media were determined using Procollagen type I C-peptide EIA Kit (Takara Bio, Shiga, Japan) or Fibronectin ELISA kit (Abcam, Cambridge, UK), respectively, per the manufacturer’s instructions. After measuring the secreted PIP or fibronectin levels, the cells were dissociated and counted using an automated cell counter (Countess II, Invitrogen, Carlsbad, CA, USA). Each measurement was divided by the number of cells and normalized. Each sample was analyzed in triplicate.

### RNA-sequencing (RNA-Seq) experiment and data analysis

The cells were treated with ultrasound at 12% amplitude for 10 s in serum-free DMEM conditions, incubated in DMEM with 10% FBS, and then harvested at 3 or 6 h post ultrasound treatment. Total RNAs were extracted using the TRIzol reagent (Invitrogen, Carlsbad, CA, USA), and poly-A containing mRNAs were purified and converted into a cDNA library for subsequent cluster generation and DNA sequencing following the instructions of the Illumina TruSeq RNA sample preparation kit (Illumina, San Diego, CA, USA). Quality control checks on raw sequence data were performed by the FastQC program. Reads for each sample were mapped to the reference genome (human hg19) by Tophat^24^. The aligned results were quantified by Cufflinks and obtained Fragments Per Kilobase of transcript per Million mapped reads (FPKM)^24^. The differentially expressed genes (DEGs) were obtained by selecting genes with log_2_-fold-changes greater than 1 among the expressed genes, which were defined by FPKM values ≥ 1 for at least one of two samples, non-treated control or ultrasound-treated cells. Using previously reported integrative statistical hypothesis testing method, which computes adjusted *P* value (*P*) by combining the two-tailed t-test and the median ratio test^25,26^, the genes with *P* < 0.05 and |log_2_-fold-changes|> 0.326 (top 1% value of null distribution obtained from 1000 times sample permutation) were selected as DEGs.

### Gene set enrichment analysis

Representative biological processes for up- and down-regulated genes were determined by performing gene set enrichment analysis using DAVID^27^. We selected Gene Ontology Biological Process with *P* value < 0.1 (default cutoff in DAVID) and gene count > 2 as the representative biological processes.

### Western blot analysis

The cells were collected at 1, 10, 20, 40, 60 and 120 min post treatment with ultrasound (12% amplitude, 10 s) and lysed with RIPA lysis buffer (Sigma, St. Louis, MO, USA) containing Protease inhibitors (Sigma). Twenty µg of cell lysates were resolved in 4–12% gradient Bio-Tris gels and transferred to nitrocellulose membranes (Invitrogen). The bands were probed using anti-p38 (Cat. No. 4511 Cell signaling Technology, BeverlyMA, USA) or anti-ERK (Cat. No. 9101 Cell signaling Technology, BeverlyMA, USA) with HRP-conjugated secondary antibodies. Western Blotting Luminol Reagent (Santa Cruz Biotechnology) was used to develop the signals and the bands were visualized using the Amersham Imager 600 system (GE Healthcare, Chicago, IL, USA).

### Quantitative real-time PCR (qRT-PCR)

The cells were exposed to ultrasound (12% amplitude, 10 s) in serum-free DMEM and incubated in DMEM with 10% FBS at 37 °C. The cells were collected at 1, 2, 3, 6, 24, 48, and 72 h post treatment with ultrasound. Total RNAs were extracted using Trizol reagent, and 2 μg of total RNAs were used to synthesize cDNA using a reverse transcriptase kit (Invitrogen). Gene expression analyses were performed using TaqMan Universal Master Mix and TaqMan Gene Expression assays (Applied Biosystems, Foster City, CA) in a 7500 Fast Real-time PCR system (Applied Biosystems) according to the manufacturer’s instruction. The 60S ribosomal protein L13a (*RPL13a*) or glucose-6-phosphate dehydrogenase (*G6PD*) were used to normalize variations of cDNA quantities synthesized from different samples. Relative differences in gene expression were calculated from Ct (threshold cycle) values as previously described^28^. The gene name and its corresponding Taqman probe used in the Taqman Gene Expression assays are as follows; *CDC25A, CDK1, CCNA2, CDK4, eIF4E, DHFR, RPL13α,* and *G6PD*.

### Three-dimensional collagen matrix contraction assay

Collagen lattices were prepared by mixing fibroblasts with a type I collagen neutralized solution. The pH of the solution for collagen (3 mg mL^−1^, type I-A cell matrix, Nitta Gelatin Inc., Osaka, Japan) was set to pH 7.4 with 5 M NaOH. The collagen solution was then diluted with DMEM with 10% FBS and added fibroblasts. The final concentrations of collagen and cells were 1 mg mL^−1^ and 2 × 10^5^ cells mL^−1^, respectively. To allow polymerization, the mixture was added to 24 well culture plate (500 μL per well) and incubated at 37 °C for 2 h. Then, the collagen lattices were incubated for 24 h in the media and stimulated with ultrasound of various amplitude. After the stimulation, the collagen lattices were released from the bottom of the culture plates using white tips and incubated in the media. Digital images of the contracting lattices were taken over 5 days at different time points with the collagen lattice areas calculated using the ImageJ software. (NIH, Bethesda, MD, USA).

### Statistical analysis

A one-way analysis of variance (one-way ANOVA) was applied to analyze the data. *P* values less than 0.05 were considered statistically significant. All data were represented as mean ± standard deviation.

## Results and discussion

### Proliferation and migration of ultrasound-activated human dermal fibroblast

**Figure 1:**
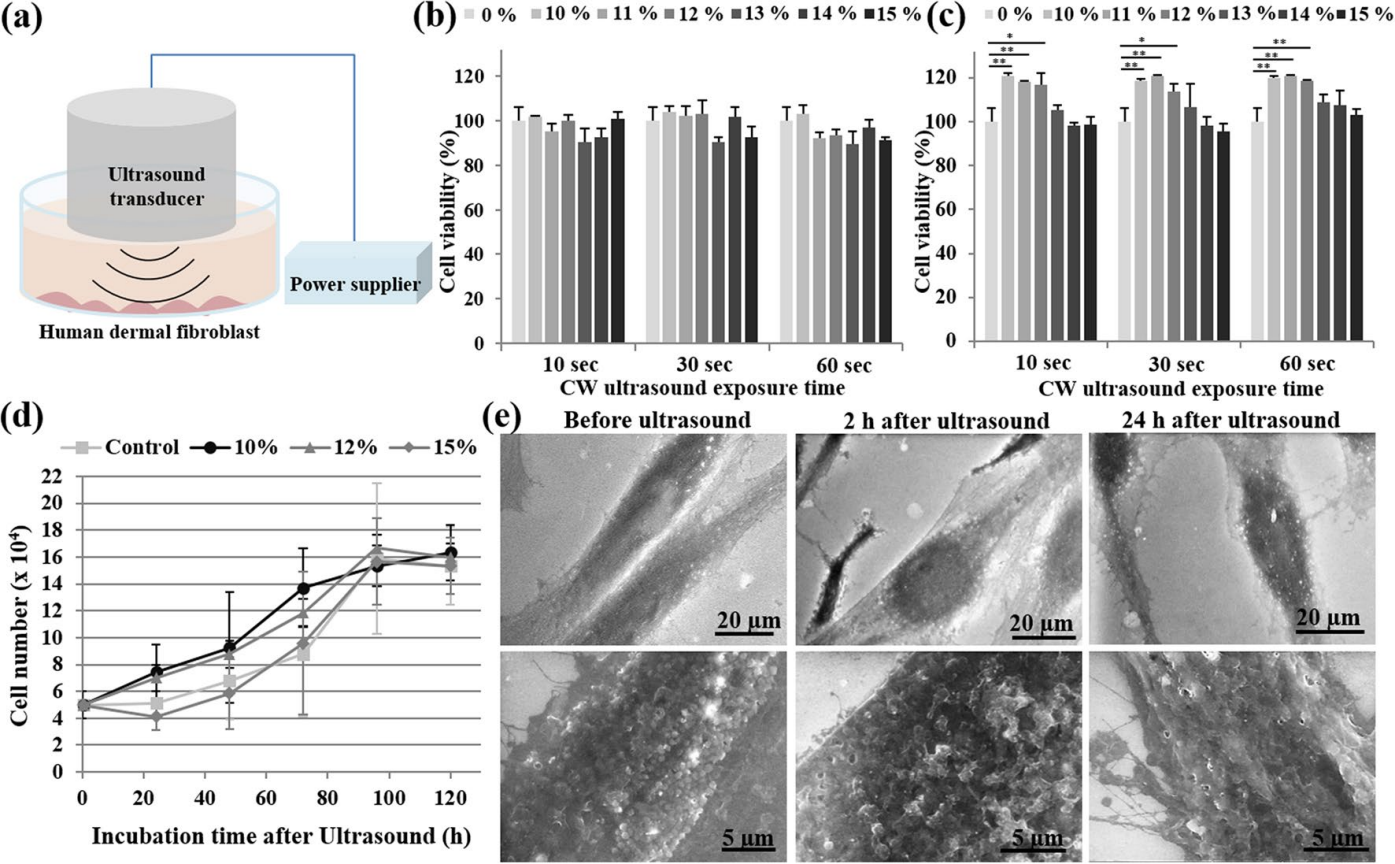
(**a**) Schematic of the set-up used for the ultrasound treatment of human dermal fibroblasts. Cell viability was measured at 10 min (**b**) or 48 h (**c**) following treatment with ultrasound of 10 to 15% amplitude for 10, 30, or 60 s. Cells treated with 0% served as a control. Data are means ± SD of three independent experiments. *P < 0.05 and **P < 0.01 compared with each control group. (**d**) Cell numbers were counted for 120 h following treatment with medium alone (control) or ultrasound with 10, 12, or 15% amplitude. Three independent experiments were performed with consistent results. (**e**) ESEM images of fibroblasts before and after treatment with 12% ultrasound for 10 s. Scale bars, 20 μm (top panel) or 5 μm (bottom panel; magnified).

To investigate whether ultrasound, as a mechanical stimulus, can activate human dermal fibroblasts without inducing cell death, fibroblasts were continuously treated with different amplitudes (10–15% of 400 W cm^−2^) of ultrasound at 20 kHz frequency for 10, 30 and 60 s. The control group consisted of treatment (0%) that the ultrasound probe was applied without activation (Fig. 1a). Cell viability was investigated at 10 min and 48 h post-ultrasound treatment.

Compared with non-activated control cells, ultrasound-treated cells did not show a significant reduction in cell viability at 10 min and 48 h post-ultrasound, even though cell loss seemed to occur slightly after 10 min by higher amplitude ultrasound treatment (Fig. 1b,c). Cells treated with amplitude of 10 to 12% exhibited a significant increase in growth at 48 h post-treatment, regardless of ultrasound treatment time (Fig. 1c). Therefore, HIUS at 10, 12, or 15% amplitude for 10 s were selected for inducing human fibroblast activation. Under this condition, the cell number of 10 and 12% ultrasound-treated fibroblasts tended to increase in a time-dependent manner compared to non-treated control cells. However, amplitude of 15% did not increase cell proliferation to the same extent (Fig. 1d). Cell proliferation in all groups reached a plateau at day 4 or 5 post-treatment, and no significant differences in proliferation were observed among the different groups at later time points (Fig. 1d). These results suggested that short-term exposure to HIUS was sufficient to promote the proliferation of fibroblasts without causing cell death, and this effect was sustained for a few days.

To observe the ultrasonic wave effect on the cell membrane, cell morphology was observed using environmental scanning electron microscopy (ESEM) after 2 and 24 h of incubation on the slide chamber following ultrasound treatment (Fig. 1e). Structural changes on the surface of the cell membranes were observed, with an apparent roughness in the membrane curvature at 2 h, and more membranous protrusion at 48 h post-treatment, compared to non-treated control cells that exhibited smooth surfaces. The change in the membrane curvature seemed to be temporary as the roughness observed at 2 h was reduced and the protrusion was increased at 48 h (Fig. 1e), which was potentially associated with behavioral changes of the cells. Consistent with the result of the cell viability assay (Fig. 1b,c), changes in the cell membrane did not lead to cell death, as demonstrated by flow cytometry analysis, which did not detect a significant proportion of apoptotic cells (Figure S2). Protrusion, the extension of cellular membranes, typically occurs in response to cellular migration signals.

**Figure 2:**
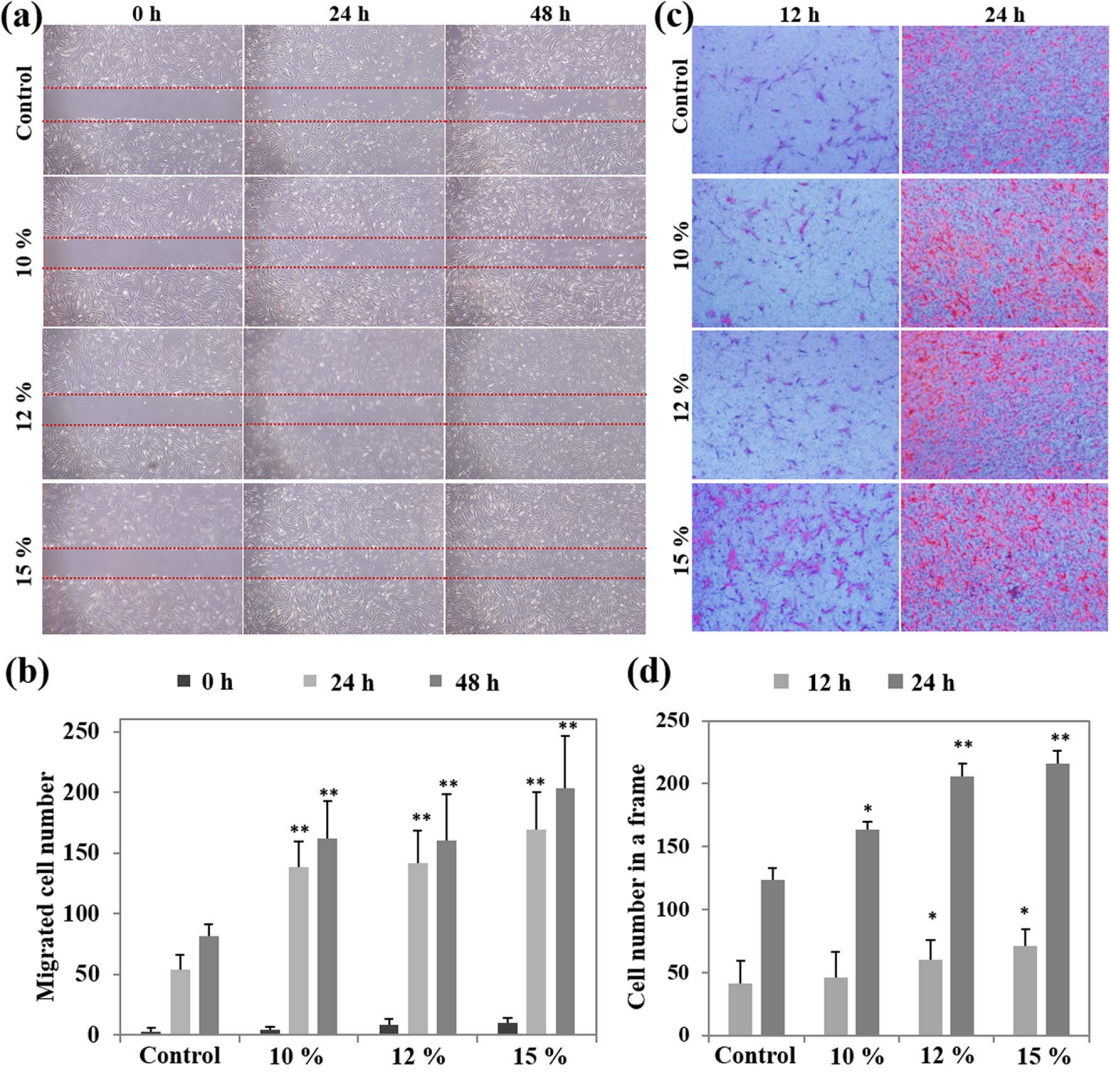
(**a**) In vitro scratch assay. Optical microscopy images were taken at 0, 24, or 48 h after treatment with 10, 12, or 15% ultrasound for 10 s. Dashed lines indicate the scratch. Control cells were treated with medium alone. (**b**) The numbers of migrated fibroblasts were quantified by direct cell counting. **P  < 0.01 compared with cell numbers at the same time in the control group. (**c**) Transwell migration assay. Fibroblasts that had migrated through the Transwell filters were fixed at 12 or 24 h after treatment with 10, 12, or 15% ultrasound for 10 s, and stained with hematoxylin. The representative images were taken under optical microscopy. (**d**) The numbers of fibroblasts in a frame were counted. *P < 0.05, **P < 0.01 compared with the cell numbers at the same time in the control group. All statistical analysis methods were one-way ANOVA.

The effect of HIUS treatment on fibroblast migration was investigated using an in vitro scratch wound assay, which was developed to measure cell migration in vitro and to mimic cell migration during wound healing in vivo to an extent^29^. The scratch in non-treated cells spontaneously closed over time; however, this effect was significantly accelerated by HIUS treatment (Fig. 2a,b). To examine whether HIUS induces the directional migration or invasion of fibroblasts, a transwell assay was performed. While all treatments resulted in a gradual increase in migration, the difference was not significant compared with the control at 12 h. HIUS treatment resulted in a significant increase in fibroblast migration at 24 h in an amplitude-dependent manner (Fig. 2c,d). These results indicated that short-term exposure to HIUS stimulated fibroblast migration in vitro, which may accelerate wound closure in vivo.

### Upregulated expression of extracellular matrix components

**Figure 3:**
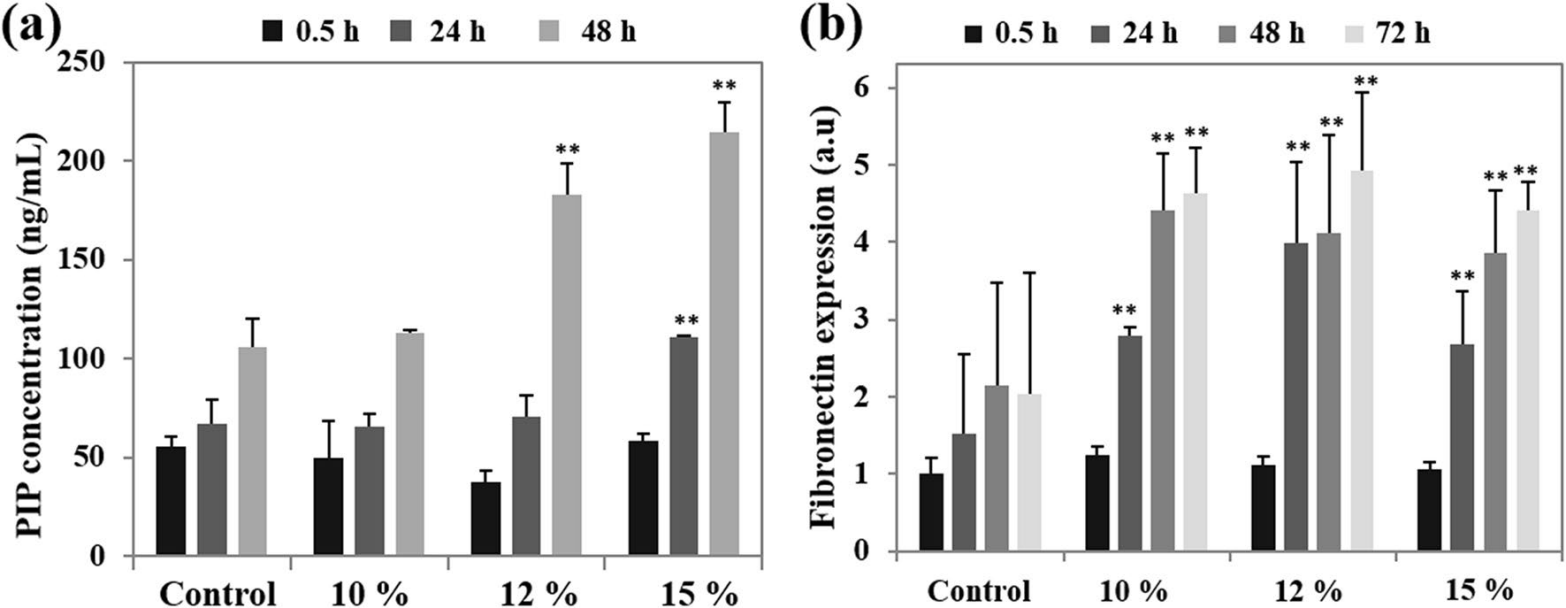
The cells were treated with media (control) or ultrasound with 10, 12, or 15% amplitude for 10 s and incubated for 0.5, 24, 48, and 72 h. Procollagen type I carboxyterminal peptide (**a**) and fibronectin (**b**) levels were determined using PIP EIA or a fibronectin ELISA kit, respectively, at each time point. Each level was normalized to cell numbers. Data are means ± SD of three independent experiments. **P < 0.01 compared with the level at the same time in the control group. All statistical analysis methods were one-way ANOVA.

Once fibroblasts move into the wounded area, they contribute to tissue regeneration by depositing fibronectin and new collagen matrix^17^. Collagen type I is the most abundant in granulation tissue, and the concentration of carboxyterminal propeptide of procollagen type I is a useful indicator of wound healing by directly reflecting collagen type I synthesis^30^. Therefore, we assessed the secreted levels of procollagen type I c-peptide (PIP) from fibroblast conditioned media after HIUS treatment. At early time points (0.5 or 24 h), HIUS treatment, except for 15%, did not stimulate new synthesis of collagen type I. However, 12 and 15% HIUS significantly increased the PIP levels at 48 h (Fig. 3a). Compared with collagen synthesis, the protein levels of fibronectin, which is another important constituent of the extracellular matrix, were increased by all treatment conditions, including 10% amplitude, from 24 to 72 h (Fig. 3b). This result suggested that HIUS stimulated fibronectin synthesis earlier and more effectively than collagen type I. Given that fibronectin interacts with specific domains on the fibroblast surfaces and is involved in several cellular activities such as cell adhesion, growth, migration and differentiation^31,32^, upregulated fibronectin expression may directly increase fibroblast proliferation and migration following HIUS treatment, and vice versa. Once fibroblasts accumulate in the wound site via migration or proliferation, they deposit collagen matrices required for granulation. Therefore, the activation of fibroblasts by HIUS treatment may be an efficient strategy to accelerate wound healing.

### Gene expression profiling

**Figure 4:**
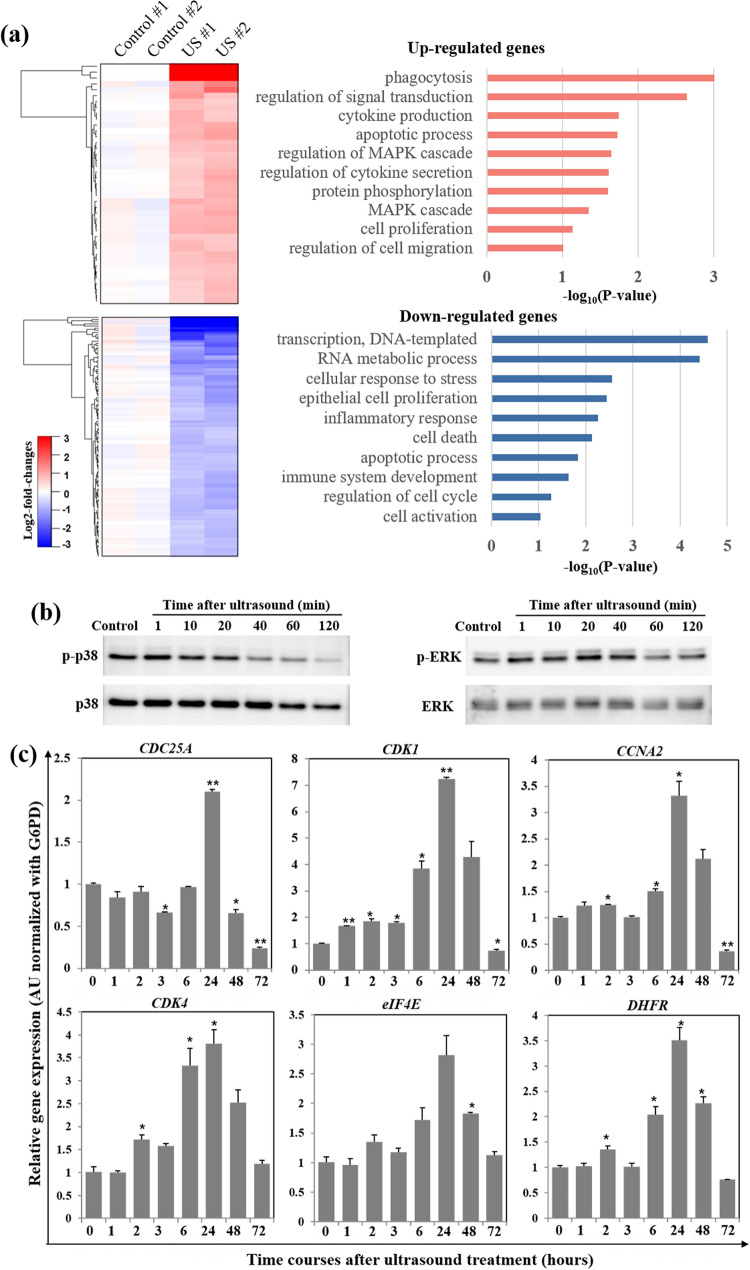
(**a**) Heat maps of upregulated and downregulated genes at 3 h post treatment with 12% ultrasound for 10 s. Differentially-expressed genes were identified using *P* < 0.05 and |log_2_-fold-change|> 0.326 as cut-off values. Biological processes associated with the upregulated (top) or downregulated (bottom) genes following ultrasound treatment. (**b**) Phosphorylated or total protein levels of p38 and ERK1/2 at the indicated time points (minutes) were determined by western blot analysis. Glucose-6-phosphate dehydrogenase (G6PD) was used for normalization. (**c**) The mRNA expression of cell proliferation-related genes was analyzed by qRT-PCR at the indicated time points (hours). *P < 0.05 and **P < 0.005 compared with the level in the control group.

To understand the molecular mechanism underlying HIUS-induced fibroblast activation and upregulated expression of extracellular matrix components such as fibronectin and collagen, we examined the gene expression profile of HIUS-treated fibroblasts using RNA-sequencing. Compared with non-treated control cells, 41 and 111 genes were upregulated and downregulated, respectively, in response to HIUS (Fig. 4a, Tables S1 and S2). Based on gene set enrichment analysis using DAVID for differentially-expressed genes ^27^, biological processes such as the MAPK cascade, cytokine production, and cell proliferation and migration were upregulated, whereas transcription-related and stress or inflammatory response-related pathways were downregulated (Fig. 4b). Of the upregulated processes, MAPK pathways mediate various cellular responses to diverse external stimuli, including mitogens and stresses, and therefore play an important role in cellular processes such as proliferation, differentiation, survival/apoptosis and tissue restoration^33,34^. For examples, in the eye, ERK and p38 MAPK pathways regulate cell cycle progression and migration of corneal cells, respectively, during wound healing^34^. We examined the activation of these MAPK pathways after HIUS treatment over time using western blot analysis and antibodies targeting the phosphorylated forms of the proteins. The phosphorylated p38 (p-p38) and ERK (p-ERK) levels were increased immediately after HIUS treatment, and the signals were sustained for several tens of minutes, after which they gradually decreased (Fig. 4b, Figure S3). This suggested that HIUS quickly activates MAPK signaling in human dermal fibroblasts. We evaluated whether the activation of MAPK pathways were accompanied by the expression of proliferation-related genes. The mRNA expression of cell cycle-associated genes including cyclin (*CCNA2*), cyclin-dependent kinases (*CDK1* and *CDK4*), phosphatase (*CDC25A*), transcription factor (*eIF4E*) and enzyme (*DHFR*) was significantly upregulated at 24 h post-treatment and downregulated thereafter (Fig. 4c). While the aforementioned genes were upregulated as early as 3 h post-HIUS treatment, a significant upregulation in cell cycle-associated gene expression occurred within 24 h after the activation of the MAPK signaling pathways that occurred within 1 h post-HIUS treatment. These results suggested that MAPK signaling pathways can be involved in HIUS-mediated fibroblast proliferation and/or migration.

### Contractility in three-dimensional collagen matrix

**Figure 5:**
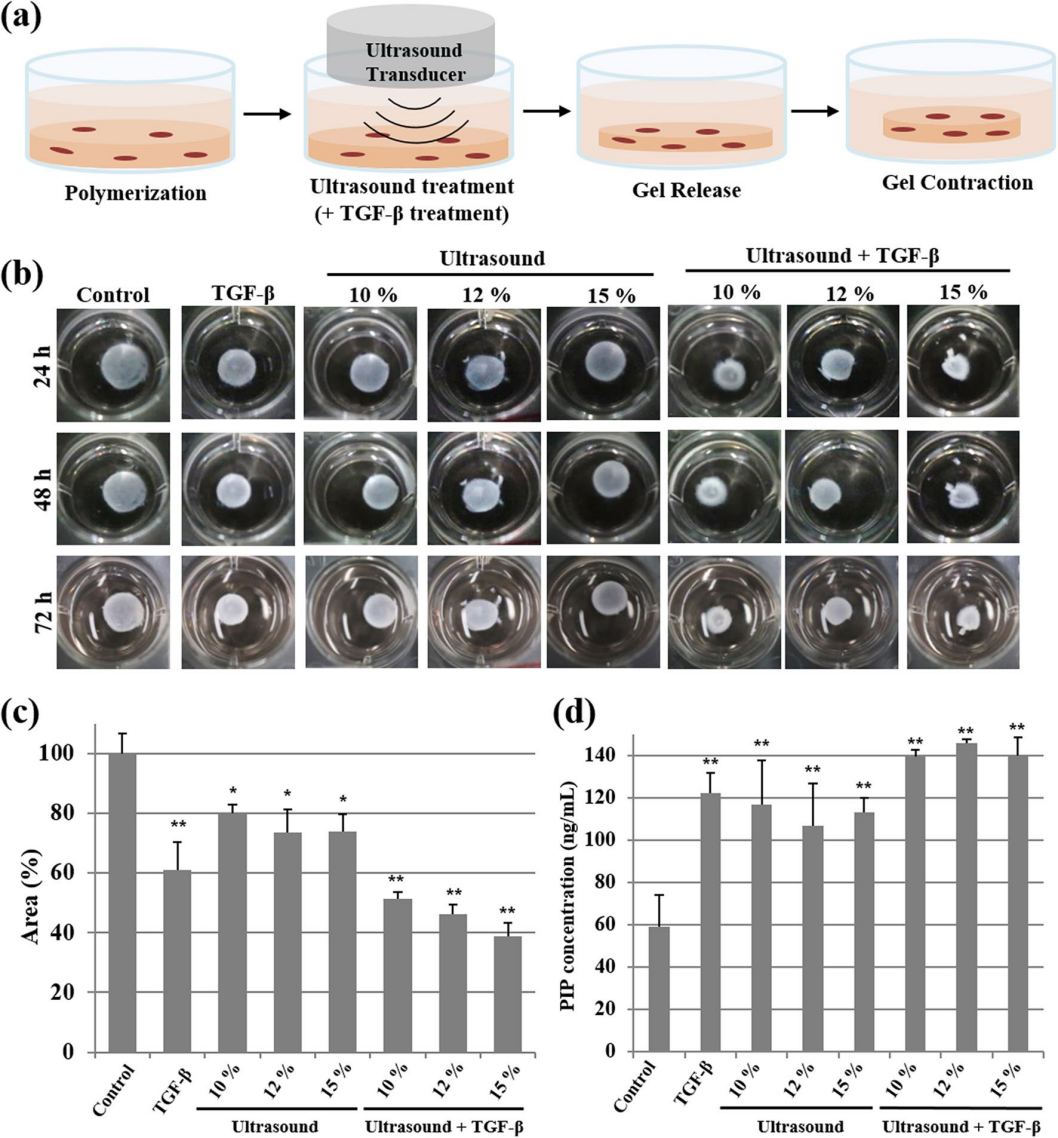
(**a**) Schematic illustration of the 3D collagen matrix contraction assay. Collagen matrices were treated with 10, 12, or 15% ultrasound for 10 s with or without TGF-β and then released. (**b**) Representative images of floating collagen matrices at 24, 48, and 72 h post-release were taken under optical microscopy. (**c**) Area of collagen matrix contracted at 72 h after treatment. (**d**) The levels of procollagen type I carboxyterminal peptide were determined in the media of the 3D collagen matrix at 72 h after each treatment using a specific PIP EIA kit. *P < 0.05, **P < 0.01 compared with the control. All statistical analysis methods were one-way ANOVA.

Contractility is an important characteristic of activated fibroblasts that is required for wound closure. To assess whether HIUS can induce the contractility of fibroblasts, we performed a cell contraction assay using the floating contraction model, in which a three-dimensional (3D) collagen matrix containing dermal fibroblasts (i.e. the dermal equivalent) were treated with HIUS with or without TGF-β, and subsequently released from the culture dish (Fig. 5a). TGF-β is involved in a number of processes in wound healing, particularly by promoting collagen synthesis and deposition, remodeling of new extracellular matrix and increasing fibroblast contractility^35,36^. As expected, compared with the area of control matrices, that of TGF β-treated matrices was significantly reduced in a time-dependent manner (Fig. 5b), indicating that the 3D collagen matrix responded to the external stimulus and TGF-β. Treatment with HIUS alone induced contraction regardless of amplitude, although the effect was relatively lower than that of TGF-β. Interestingly, when cells were treated with HIUS and TGF-β sequentially, the contractility of fibroblasts was notably increased compared with fibroblasts treated with either HIUS or TGF-β alone (Fig. 5b,c and Figure S5). These results suggested that short-term exposure to HIUS may be sufficient to increase the contractility of fibroblasts; however, more interestingly, it can maximize the effects of other reagents such as TGF-β, for wound healing. Similar to the contraction, the PIP levels in the conditioned media were increased by TGF-β or HIUS treatment compared with the non-treated control, but was further increased with TGF-β and HIUS co-treatment (Fig. 5d), indicating that fibroblasts embedded in a 3D collagen matrix synthesize new collagen type I upon exposure to TGF-β or HIUS. Previously, it was suggested that TGF-β increases contractility by inducing the differentiation of fibroblasts into myofibroblasts, and stimulating the production of extracellular matrix^36–38^. There is a possibility that HIUS can induce the differentiation of fibroblasts into myofibroblasts, as HIUS has successfully been applied for the transdifferentiation of human fibroblasts to multipotent stem/progenitor cells^13,39^. The increased production of extracellular matrix components such as collagen and fibronectin may be directly associated with collagen matrix contraction, as they are required for myofibroblast-mediated contraction^40^.

Several approaches, including the topical application of growth factors, skin substitutes or the injection of fibroblasts or stem cells, in addition to debridement, antibiotic or pressure treatment, have been developed to treat wounds resulting from burns, traumatic injuries and diabetes, where healing and scarring is delayed. However, the outcome is often unsatisfactory due to enzymatic degradation or cost. Therefore, the development of effective alternatives to promote wound healing is urgently required. The present study demonstrated that HIUS enhanced wound healing in vitro, suggesting that HIUS may be used as a non-invasive option for wound management, and to maximize the effect of other reagents, such as growth factors, by improving their delivery to tissues.

## Conclusions

In summary, we demonstrated that HIUS facilitated wound closure in vitro and increased contraction of a dermal equivalent by promoting the proliferation and migration of human dermal fibroblasts, subsequently increasing the production of extracellular matrix components. Given that the aforementioned processes constitute essential steps for wound healing and tissue repair, short-term exposure to HIUS may be an effective alternative for the treatment of various wound types and to improve tissue regeneration.

## Supplementary Information

Supplementary Information

## References

[1] Lee JY (2015). Nanoparticle-loaded protein-polymer nanodroplets for improved stability and conversion efficiency in ultrasound imaging and drug delivery. Adv. Mater..

[2] Sirsi SR, Borden MA (2014). State-of-the-art materials for ultrasound-triggered drug delivery. Adv. Drug Deliv. Rev..

[3] Daeschler SC (2018). Ultrasound and shock-wave stimulation to promote axonal regeneration following nerve surgery: A systematic review and meta-analysis of preclinical studies. Sci. Rep..

[4] Hannemann PF, Mommers EH, Schots JP, Brink PR, Poeze M (2014). The effects of low-intensity pulsed ultrasound and pulsed electromagnetic fields bone growth stimulation in acute fractures: A systematic review and meta-analysis of randomized controlled trials. Arch. Orthop. Trauma Surg..

[5] Jia XL, Chen WZ, Zhou K, Wang ZB (2005). Effects of low-intensity pulsed ultrasound in repairing injured articular cartilage. Chin. J. Traumatol..

[6] Takakura Y (2002). Low-intensity pulsed ultrasound enhances early healing of medial collateral ligament injuries in rats. J. Ultrasound Med..

[7] Chen YJ (2003). Pertussis toxin-sensitive Galphai protein and ERK-dependent pathways mediate ultrasound promotion of osteogenic transcription in human osteoblasts. FEBS Lett..

[8] Zhou S (2004). Molecular mechanisms of low intensity pulsed ultrasound in human skin fibroblasts. J. Biol. Chem..

[9] Kusuyama J (2014). Low intensity pulsed ultrasound (LIPUS) influences the multilineage differentiation of mesenchymal stem and progenitor cell lines through ROCK-Cot/Tpl2-MEK-ERK signaling pathway. J. Biol. Chem..

[10] Nakao J (2014). Low-intensity pulsed ultrasound (LIPUS) inhibits LPS-induced inflammatory responses of osteoblasts through TLR4-MyD88 dissociation. Bone.

[11] Xia P (2017). TGF-beta1-induced chondrogenesis of bone marrow mesenchymal stem cells is promoted by low-intensity pulsed ultrasound through the integrin-mTOR signaling pathway. Stem Cell Res. Ther..

[12] Xin Z, Lin G, Lei H, Lue TF, Guo Y (2016). Clinical applications of low-intensity pulsed ultrasound and its potential role in urology. Transl. Androl. Urol..

[13] Lee YS (2017). An ultra-effective method of generating extramultipotent cells from human fibroblasts by ultrasound. Biomaterials.

[14] Roper JA (2015). Ultrasonic stimulation of mouse skin reverses the healing delays in diabetes and aging by activation of Rac1. J. Invest. Dermatol..

[15] Zhou X (2016). Improved human bone marrow mesenchymal stem cell osteogenesis in 3D bioprinted tissue scaffolds with low intensity pulsed ultrasound stimulation. Sci. Rep..

[16] Shaw TJ, Martin P (2009). Wound repair at a glance. J. Cell. Sci..

[17] Shaw TJ, Martin P (2016). Wound repair: A showcase for cell plasticity and migration. Curr. Opin. Cell Biol..

[18] Blacklow SO (2019). Bioinspired mechanically active adhesive dressings to accelerate wound closure. Sci. Adv..

[19] Fitzgerald O’Connor EJ (2012). Histone deacetylase 2 is upregulated in normal and keloid scars. J. Invest. Dermatol..

[20] Glenisson W, Castronovo V, Waltregny D (2007). Histone deacetylase 4 is required for TGFbeta1-induced myofibroblastic differentiation. Biochim. Biophys. Acta.

[21] Driskell RR (2013). Distinct fibroblast lineages determine dermal architecture in skin development and repair. Nature.

[22] Achterberg VF (2014). The nano-scale mechanical properties of the extracellular matrix regulate dermal fibroblast function. J. Invest. Dermatol..

[23] Li W (2007). Extracellular heat shock protein-90alpha: Linking hypoxia to skin cell motility and wound healing. EMBO J..

[24] Trapnell C (2012). Differential gene and transcript expression analysis of RNA-seq experiments with TopHat and Cufflinks. Nat. Protoc..

[25] Hwang D (2005). A data integration methodology for systems biology. Proc. Natl. Acad. Sci. U. S. A..

[26] Jeong HS (2017). Transcriptional regulatory networks underlying the reprogramming of spermatogonial stem cells to multipotent stem cells. Exp. Mol. Med..

[27] da Huang W, Sherman BT, Lempicki RA (2009). Systematic and integrative analysis of large gene lists using DAVID bioinformatics resources. Nat. Protoc..

[28] Livak KJ, Schmittgen TD (2001). Analysis of relative gene expression data using real-time quantitative PCR and the 2−ΔΔCT method. Methods.

[29] Liang CC, Park AY, Guan JL (2007). In vitro scratch assay: A convenient and inexpensive method for analysis of cell migration in vitro. Nat. Protoc..

[30] Haukipuro K, Melkko J, Risteli L, Kairaluoma M, Risteli J (1991). Synthesis of type I collagen in healing wounds in humans. Ann. Surg..

[31] Bitterman PB, Rennard SI, Adelberg S, Crystal RG (1983). Role of fibronectin as a growth factor for fibroblasts. J. Cell. Biol..

[32] Pankov R, Yamada KM (2002). Fibronectin at a glance. J. Cell Sci..

[33] Pearson G (2001). Mitogen-activated protein (MAP) kinase pathways: Regulation and physiological functions. Endocr. Rev..

[34] Yao B (2017). MAPK signaling pathways in eye wounds: Multifunction and cooperation. Exp. Cell Res..

[35] Liu XD (2001). Persistence of TGF-beta1 induction of increased fibroblast contractility. Vitro Cell Dev. Biol. Anim..

[36] Lijnen P, Petrov V, Fagard R (2003). Transforming growth factor-beta 1-mediated collagen gel contraction by cardiac fibroblasts. J. Renin. Angiotensin Aldosterone Syst..

[37] Leask A, Abraham DJ (2004). TGF-beta signaling and the fibrotic response. FASEB J..

[38] Carthy JM (2018). TGFbeta signaling and the control of myofibroblast differentiation: Implications for chronic inflammatory disorders. J. Cell Physiol..

[39] Lee YS (2018). Exosome-mediated ultra-effective direct conversion of human fibroblasts into neural progenitor-like cells. ACS Nano.

[40] Kattan WM (2017). Myofibroblast-mediated contraction. J. Coll. Phys. Surg. Pak..

