# Supplementary material for: Non pharmacological high-intensity ultrasound treatment of human dermal fibroblasts to accelerate wound healing
Source: Sci Rep. 2021 Jan 28;11:2465. doi: 10.1038/s41598-021-81878-1 (PMC7844265; doi:10.1038/s41598-021-81878-1)
Supplement: Supplementary file 1 — Supplementary Information [file 41598_2021_81878_MOESM1_ESM.docx]

**Supporting Information**

Non pharmacological high-intensity ultrasound treatment of human dermal fibroblasts to accelerate wound healing

Jeong Yu Lee**^1,*^**, Daejin Min**^1^**, Wanil Kim**^1^**, Bum Ho Bin**^1^**, Kyu Han Kim**^1^**, Eun-Gyung Cho**^1, *^**

**^1^**Basic Research & Innovation Division, R&D Unit, AmorePacific Corporation, 1920 Yonggu-daero, Giheung-gu, Yongin-si, Gyeonggi-do, Republic of Korea

*To whom all correspondence should be addressed. E-mail: [jeongyu@amorepacific.com](mailto:jeongyu@amorepacific.com) or [egcho@amorepacific.com](mailto:egcho@amorepacific.com)


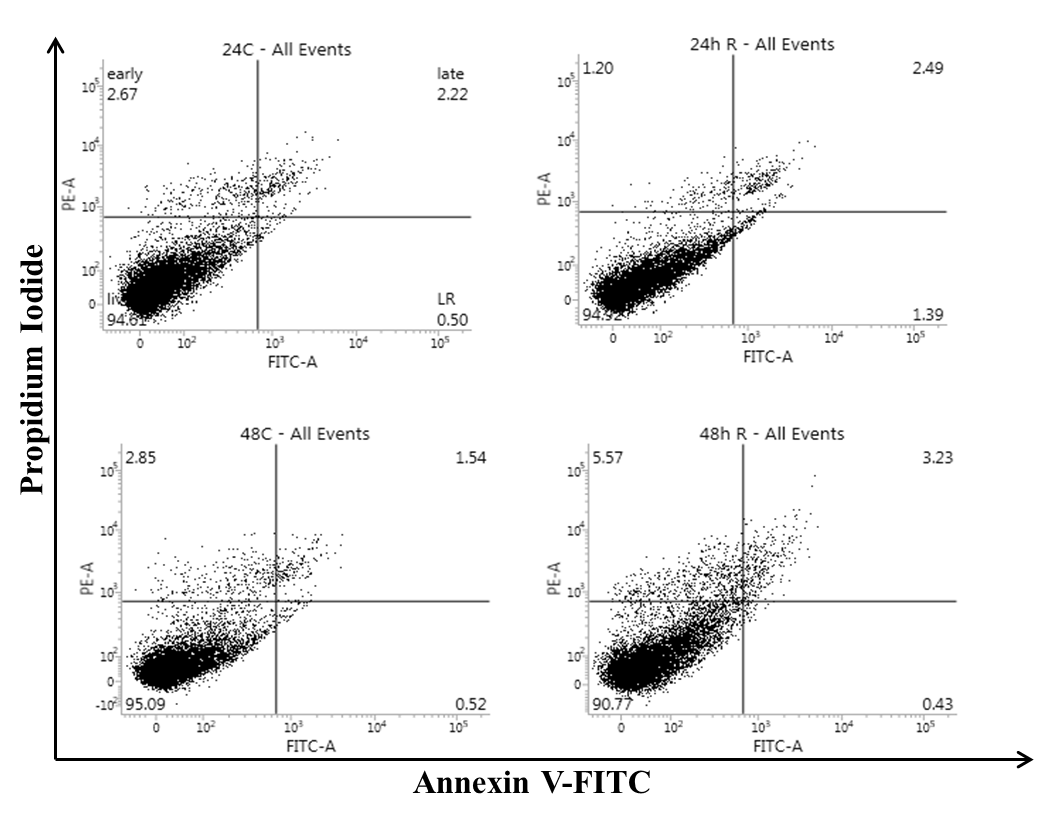


**Figure S1.** Flow cytometric analysis using Annexain V-FITC/PI staining for apoptosis.


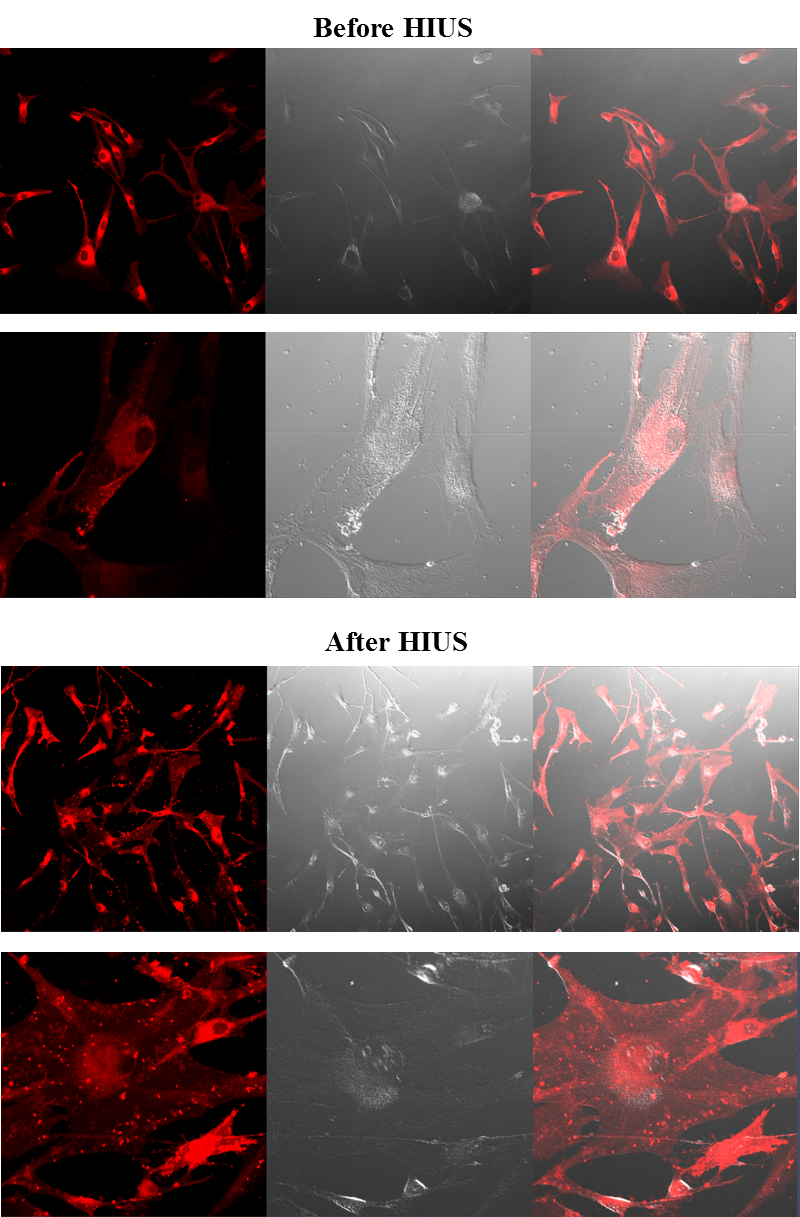


**Figure S2.** Confocal fluorescence microscopic images of fibroblast stained with PI. Images were taken at before and 24 h after treatment.

**Table S1**. The list of upregulated DEGs.

| **Term** | **Genes** |
| --- | --- |
| Phagocytosis | LRP1, ADORA2A, F2RL1, ABCA1, CD14 |
| Regulation of signal transduction | IRAK2, ICAM1, LRP1, ADORA2A, RNF152, RGS4, EPM2A, F2RL1, ABCA1, PMEPA1, CD14, TRIB2 |
| Cytokine production | CD274, F2RL1, ABCA1, CD14, TRIB2 |
| Apoptotic process | ICAM1, LRP1, ADORA2A, RNF152, CD274, BCL2A1, CD14, DHCR24 |
| Regulation of MAPK cascade | IRAK2, ICAM1, RGS4, F2RL1, TRIB2 |
| Regulation of cytokine secretion | CD274, F2RL1, CD14 |
| Protein phosphorylation | ST3GAL1, IRAK2, ICAM1, ADORA2A, RGS4, F2RL1, PMEPA1, TRIB2 |
| MAPK cascade | IRAK2, ICAM1, RGS4, F2RL1, TRIB2 |
| Cell proliferation | EDNRA, LRP1, ADORA2A, PTGES, CD274, F2RL1, DHCR24 |
| Regulation of cell migration | ICAM1, LRP1, CD274, F2RL1 |

**Table S2.** The list of downregulated DEGs.

| **Term** | **Genes** |
| --- | --- |
| RNA metabolic process | NFKBID, TRPV1, EZH1, RBM6, INTS3, NSUN5P1, FOS, OSR2, DDX11, HMOX1, SRRM2, MEG3, MYC, CYR61, RFX8, EGR1, ZFP36, PAN2, UCN, EGR2, CREBZF, NR4A1, FOSB, SNAI1, CAPN3, CCNL2, STAT2, SUGP2, BTG2, ID2, ZNF692, HES4, ZGLP1, HOXD4, VGLL3, KLF2, CRYM |
| Transcription,  DNA-templated | NFKBID, TRPV1, EZH1, INTS3, FOS, OSR2, DDX11, HMOX1, MEG3, MYC, CYR61, RFX8, EGR1, ZFP36, UCN, EGR2, CREBZF, NR4A1, FOSB, SNAI1, CAPN3, CCNL2, STAT2, BTG2, ID2, ZNF692, HES4, ZGLP1, HOXD4, VGLL3, KLF2, CRYM |
| Cell death | ZFP36, EGR1, UCN, NFKBID, GSDMB, TRPV1, NR4A1, SNAI1, CAPN3, DHRS2, CCR7, BTG2, DUSP1, HMOX1, GADD45B, MYC, CYR61 |
| Apoptotic process | ZFP36, UCN, NFKBID, TRPV1, NR4A1, SNAI1, CAPN3, DHRS2, CCR7, BTG2, DUSP1, HMOX1, GADD45B, MYC, CYR61 |
| Inflammatory response | ZFP36, FOS, UCN, CCR7, NFKBID, TRPV1, HMOX1, CXCL2, PLA2G4B |
| Immune system development | ZFP36, EGR1, DHRS2, FOS, CCR7, ID2, NFKBID, KLF2, MYC |
| Regulation of cell cycle | DUSP1, BTG2, ID2, DDX11, NR4A1, INTS3, GADD45B, MYC, CCNL2 |
| Cellular response to stress | ZFP36, EGR1, TRPV1, INTS3, SNAI1, CAPN3, DHRS2, FOS, CCR7, DDX11, ID2, BTG2, HMOX1, KLF2, GADD45B, PLA2G4B, MYC |
| Cell activation | EGR1, DHRS2, CCR7, ID2, NFKBID, TRPV1, HMOX1, CAPN3 |
| Epithelial cell proliferation | ZFP36, OSR2, ID2, HMOX1, IQGAP3, NR4A1, MYC |


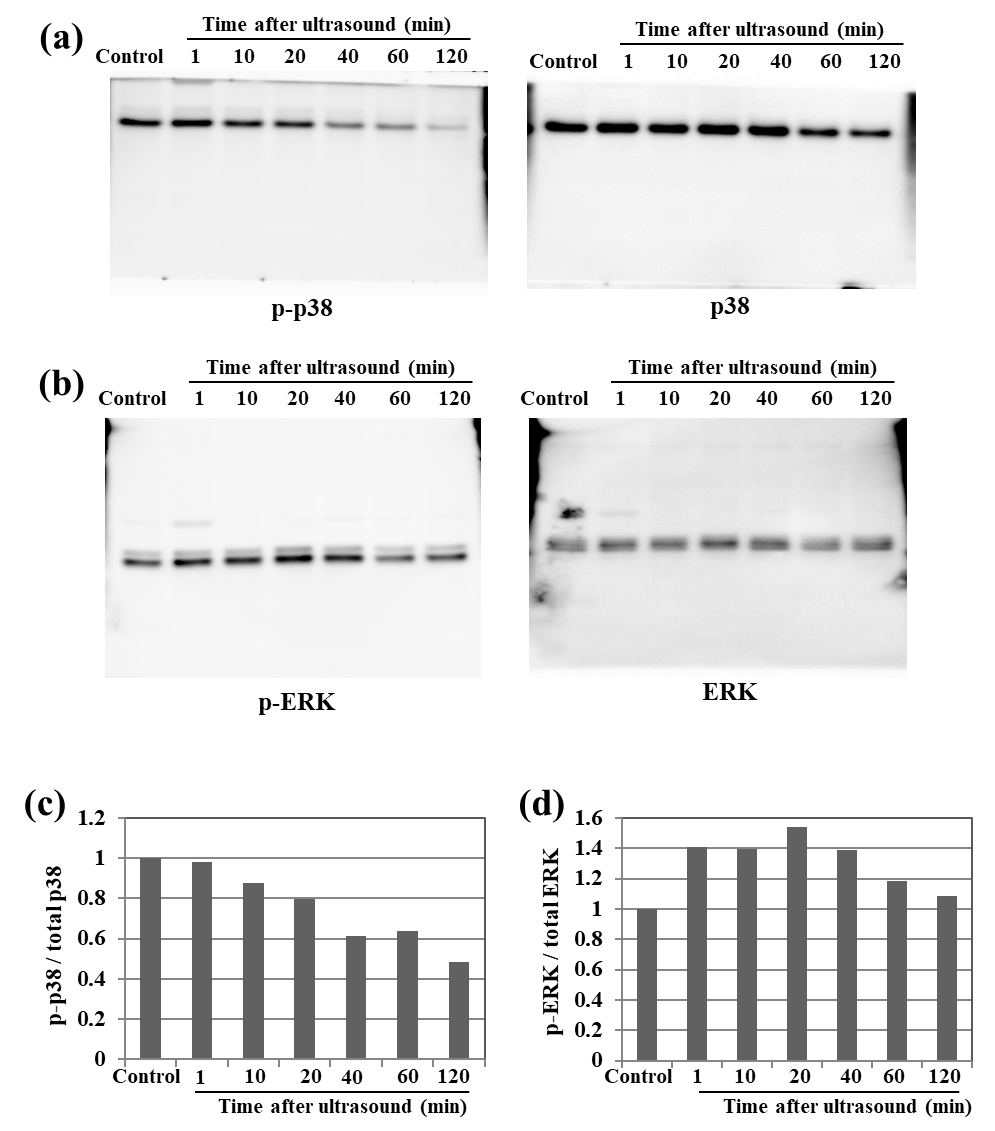


**Figure S3**. Full-length images of phosphorylated or total protein levels of p38 (a) and ERK1/2 (b) at the indicated time points (minutes) were determined by western blot analysis. All images were analyzed under the same condition. Ratio of phospho-p38 and total p38 (c) and phospho-ERK and total ERK (d) in absolute values.


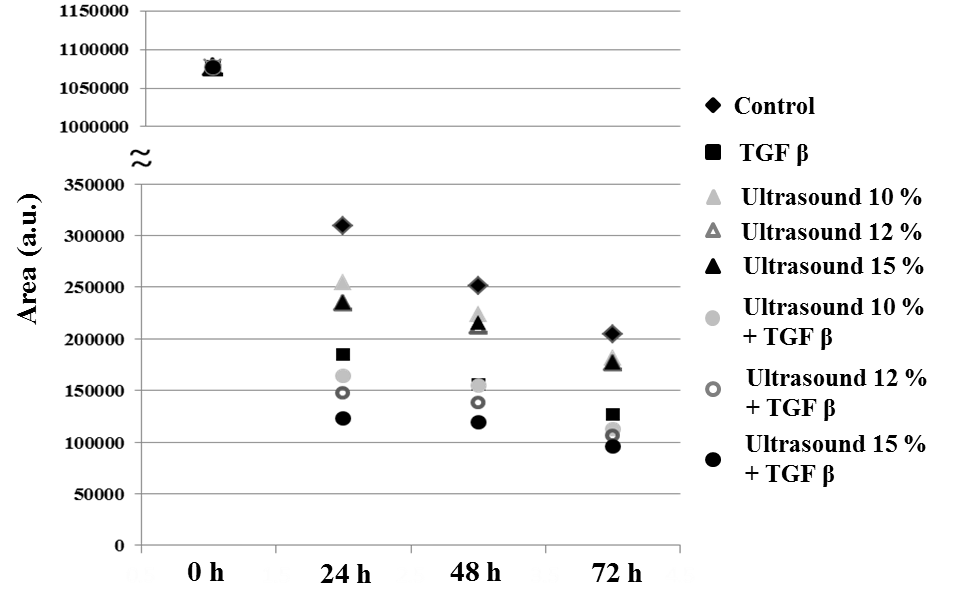


**Figure S4.** Change of the area contracted by fibroblast in 3D collagen matrix.


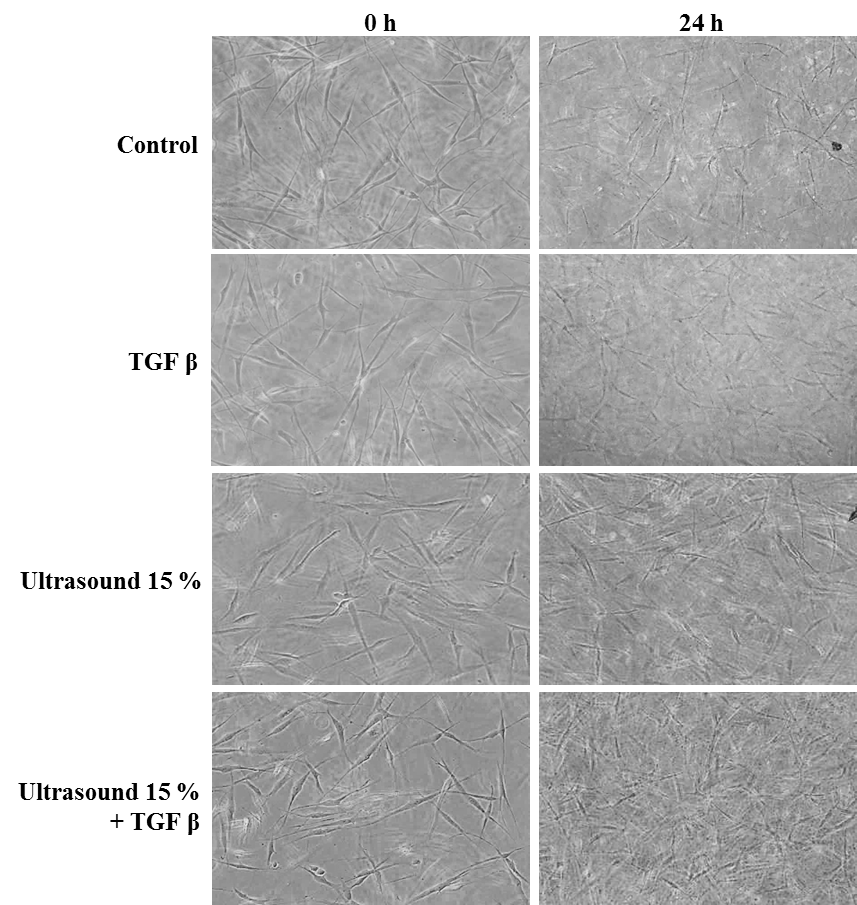


**Figure S5**. Optical microscope images of fibroblast in 3D collagen matrix. Images were taken at 0 and 24 h after HIUS treatment.
